# Revised Diagnostic Algorithm for Sarcopenic Obesity in Japan After the Asian Working Group for Sarcopenia 2025 Consensus Update (JWGSO 2026)

**DOI:** 10.1111/ggi.70815

**Published:** 2026-09-14

**Authors:** Kojiro Ishii, Wataru Ogawa, Yutaka Kimura, Toru Kusakabe, Ryo Miyazaki, Kiyoshi Sanada, Noriko Satoh‐Asahara, Kentaro Ikeue, Yoshifumi Tamura, Kohjiro Ueki, Hidetaka Wakabayashi, Yuya Watanabe, Minoru Yamada, Hidenori Arai

**Affiliations:** ^1^ Faculty of Health and Sports Science Doshisha University Kyotanabe Kyoto Japan; ^2^ Section of Metabolic Diseases, Division of Translational Science Kobe University Graduate School of Medicine Kobe Hyogo Japan; ^3^ Health Science Center Kansai Medical University Hirakata Osaka Japan; ^4^ Department of Endocrinology, Metabolism, and Hypertension Research, Clinical Research Institute National Hospital Organization Kyoto Medical Center Kyoto Japan; ^5^ Faculty of Human Sciences Shimane University Matsue Shimane Japan; ^6^ Faculty of Sport and Health Science Ritsumeikan University Kusatsu Shiga Japan; ^7^ National Center for Geriatrics and Gerontology Obu Aichi Japan; ^8^ Sportology Center Juntendo University Graduate School of Medicine Tokyo Japan; ^9^ Department of Metabolism and Endocrinology Juntendo University Graduate School of Medicine Tokyo Japan; ^10^ Diabetes Research Center National Center Institute of Global Health and Medicine, Japan Institute for Health Security Tokyo Japan; ^11^ Department of Rehabilitation Medicine Tokyo Women's Medical University Hospital Tokyo Japan; ^12^ Faculty of Sport Study Biwako Seikei Sport College Otsu Shiga Japan; ^13^ Faculty of Human Sciences University of Tsukuba Tsukuba Ibaraki Japan

To the Editor,

1

The Japanese Working Group on Sarcopenic Obesity (JWGSO), jointly established by the Japan Society for the Study of Obesity and the Japanese Association on Sarcopenia and Frailty, previously published consensus diagnostic criteria for sarcopenic obesity in Japan [1]. The statement defined sarcopenic obesity as the coexistence of obesity and sarcopenia and proposed a two‐step algorithm: screening by sarcopenia‐related measures and waist circumference or body mass index (BMI), followed by diagnostic assessment of sarcopenia and adiposity [1]. The diagnostic component included low handgrip strength, the five‐times chair stand test, low BMI‐corrected limb skeletal muscle mass, and obesity defined by visceral fat area or body fat percentage [1].

The Asian Working Group for Sarcopenia (AWGS) 2025 consensus update now provides a new framework for sarcopenia diagnosis and muscle health promotion in Asian populations [2]. It harmonizes with the Global Leadership Initiative in Sarcopenia by defining sarcopenia as concurrent low muscle mass and low muscle strength, while categorizing physical performance measures, including the five‐times chair stand test, as outcome measures rather than diagnostic criteria [2, 3]. AWGS 2025 also expands muscle health assessment into middle age and provides age‐stratified cutoffs for adults aged 50–64 years and ≥ 65 years [2].

In response, JWGSO has revised the Japanese diagnostic algorithm for sarcopenic obesity (JWGSO 2026; Figure 1). The revision has three principal changes. First, the previous age restriction of 40 years or older and younger than 75 years, originally set because evidence was limited in adults aged ≥ 75 years, has been removed [1]. This aligns with the AWGS 2025 life‐course concept and should facilitate evidence generation in this age group [2]. Clinical judgment remains essential in very old adults.

**FIGURE 1:**
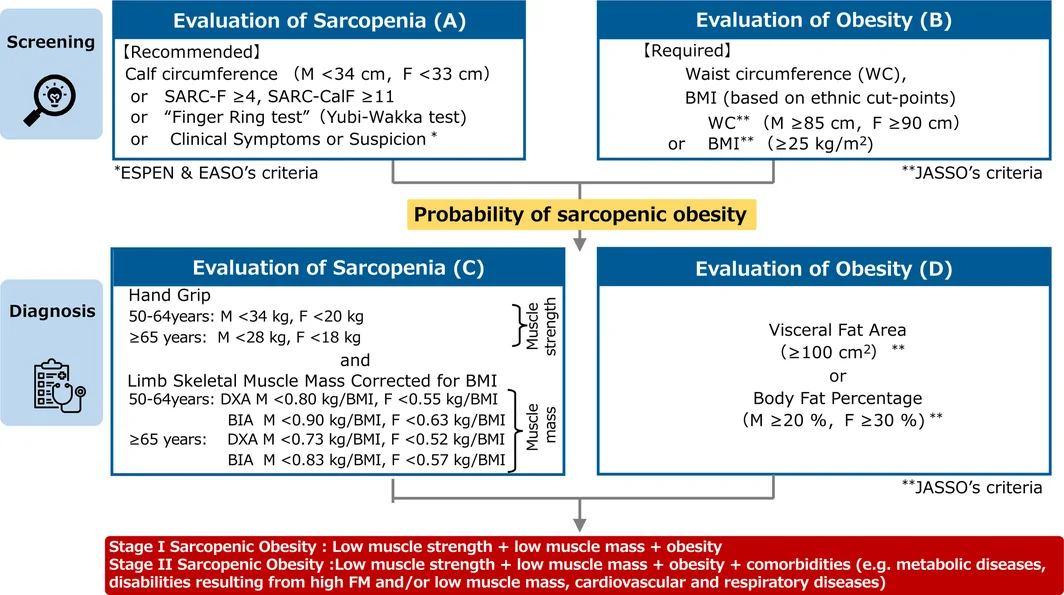
Revised diagnostic algorithm for sarcopenic obesity in Japan (JWGSO 2026). Screening for sarcopenia is recommended and includes calf circumference, SARC‐F, SARC‐CalF, the Yubi‐Wakka test, and clinical symptoms or suspicion. Screening for obesity is required and uses waist circumference or body mass index based on Japanese criteria. Diagnosis requires low handgrip strength, low limb skeletal muscle mass corrected for body mass index, and obesity defined by visceral fat area or body fat percentage. BIA, bioelectrical impedance analysis; BMI, body mass index; DXA, dual‐energy X‐ray absorptiometry; ESPEN, European Society for Clinical Nutrition and Metabolism; EASO, European Association for the Study of Obesity; JASSO, Japan Society for the Study of Obesity; JWGSO, Japanese Working Group on Sarcopenic Obesity; WC, waist circumference.

Second, the five‐times chair stand test has been removed from the diagnostic criteria for sarcopenia. Diagnosis in the revised JWGSO algorithm now requires both low muscle strength and low muscle mass. Low muscle strength is assessed by handgrip strength, using AWGS 2025 cutoffs of < 34 kg in men and < 20 kg in women aged 50–64 years, and < 28 kg in men and < 18 kg in women aged ≥ 65 years [2]. Although AWGS 2025 provides no handgrip‐strength cutoffs for individuals aged < 50 years, the 50–64‐year cutoffs may be applied provisionally; further evidence is needed for this age group. Physical performance measures remain useful for risk stratification, monitoring intervention effects, and predicting outcomes, but are not required for diagnosis.

Third, BMI‐corrected limb skeletal muscle mass cutoffs have been updated according to AWGS 2025 and are applied to both dual‐energy X‐ray absorptiometry (DXA) and bioelectrical impedance analysis (BIA) [2]. Because sarcopenic obesity is evaluated in the setting of excess adiposity, JWGSO selected BMI‐corrected limb skeletal muscle mass as the sole muscle‐mass index. Visser et al. showed that low‐muscle‐mass prevalence by height‐, weight‐, or BMI‐adjusted ratios remained BMI‐dependent in older US and Japanese samples, indicating that muscle‐mass cutoffs should account for BMI [4]. For adults aged 50–64 years, the DXA cutoffs are < 0.80 kg/BMI in men and < 0.55 kg/BMI in women, and the BIA cutoffs are < 0.90 kg/BMI in men and < 0.63 kg/BMI in women. For adults aged ≥ 65 years, the DXA cutoffs are < 0.73 kg/BMI in men and < 0.52 kg/BMI in women, and the BIA cutoffs are < 0.83 kg/BMI in men and < 0.57 kg/BMI in women.

The obesity component remains based on Japanese obesity criteria [5]. Screening requires high waist circumference or BMI, using Japanese cutoffs of waist circumference ≥ 85 cm in men and ≥ 90 cm in women, or BMI ≥ 25 kg/m^2^. Diagnostic confirmation of obesity is based on visceral fat area ≥ 100 cm^2^ or body fat percentage ≥ 20% in men and ≥ 30% in women. The sarcopenia screening step remains recommended but not mandatory, and includes calf circumference, SARC‐F, SARC‐CalF, the Yubi‐Wakka test and clinical symptoms or suspicion [1, 6].

The revised JWGSO 2026 algorithm therefore aligns Japanese criteria for sarcopenic obesity with current Asian sarcopenia criteria while preserving Japan‐specific obesity assessment. Stage I sarcopenic obesity is low muscle strength, low muscle mass, and obesity; Stage II additionally includes comorbidities, including metabolic, disability‐related, cardiovascular, or respiratory diseases. Future studies should validate the algorithm across age strata, evaluate its prognostic value, examine DXA–BIA comparability, and clarify how muscle‐health concepts can be incorporated into sarcopenic obesity diagnosis. Pathophysiological studies should examine muscle quality, ectopic fat, inflammation, and muscle‐adipose‐organ crosstalk to distinguish sarcopenic obesity from simple coexistence and determine whether mechanisms differ between trajectories from nonsarcopenic obesity to sarcopenic obesity and from nonobese sarcopenia to sarcopenic obesity.

## Author Contributions

K.I. drafted the manuscript. All authors contributed to the consensus process, critically revised the manuscript for important intellectual content, and approved the final version.

## Funding

The authors have nothing to report.

## Ethics Statement

The authors have nothing to report.

## Consent

The authors have nothing to report.

## Conflicts of Interest

The authors declare no conflicts of interest.

## Data Availability

Data sharing not applicable to this article as no datasets were generated or analyzed during the current study.
